# Primary Biliary Cholangitis With Pulmonary Manifestations and Concurrent Enterococcus Pneumonia: A Diagnostic Challenge Resembling Sarcoidosis or Silicosis

**DOI:** 10.7759/cureus.59160

**Published:** 2024-04-27

**Authors:** Muthanna Mohammed Hasan Al-Ghuraibawi, Parinitha Neravanda Prasad, Uma Gupta, Pulok Roy, Pwint Phyu Hlaing

**Affiliations:** 1 Internal Medicine, One Brooklyn Health/Interfaith Medical Center, New York, USA; 2 Internal Medicine, Adichunchanagiri Institute of Medical Sciences, Nagamangala, IND; 3 Internal Medicine, University of Medicine (1), Yangon, MMR

**Keywords:** elevated direct bilirubin, antimitochondrial antibody, enterococcus feacalis, african american male, male primary biliary cholangitis

## Abstract

Primary biliary cholangitis (PBC) is common in females during middle age, presenting with fatigue and itching. In our case, an African-American male patient presented with abdominal pain, vomiting, fatigue, and lung manifestations such as interstitial lung disease, granulomatous lung disease, and pulmonary hypertension. In our case, the patient reported abdominal pain and fatigue with abnormal chest X-ray findings (bilateral pulmonic nodular lesion with calcifications), which mimicked silicosis/sarcoidosis lung findings such as bronchiectasis and parenchymal nodules. We diagnosed PBC as there was an absence of extrahepatic biliary obstruction and the presence of antimitochondrial antibodies (AMA) at a titer of 1:40 or higher. Bronchoalveolar lavage was performed due to the suspicion of interstitial lung disease and sarcoidosis, which was inconclusive but revealed enterococcus faecalis organisms. Initial antibiotic response heightens suspicion of infection, not colonization, leading to the diagnosis of enterococcal pneumonia. In our case, the diagnosis was made using clinical and laboratory criteria, and treatment with Ursodeoxycholic acid was opted for without resorting to more expensive and invasive tests like magnetic resonance cholangiopancreatography (MRCP) and endoscopic retrograde cholangiopancreatography (ERCP). In summary, this case report presented the unique diagnostic challenges that will aid clinicians in considering a broad range of differential diagnoses and management plans.

## Introduction

Primary biliary cholangitis (PBC) is a condition that primarily affects the small bile ducts within the liver, leading to inflammation and, eventually, scarring. Antimitochondrial antibodies (AMA) serve as the serologic hallmark of PBC, demonstrating 95% sensitivity and 98% specificity in identifying PBC patients [1,2].

Non-occupational exposure to crystalline silica occurs in hilly areas through desert dust and sand storms, and our patient has similar environmental exposure [3,4]. On the other hand, sarcoidosis can present with similar imaging findings, such as pulmonary reticular and nodular opacities, commonly observed in African Americans within the age range of 20 to 60 years, similar to the characteristics observed in our case [5].

Our extensive literature search supports the findings of organisms that could be related to biliary pathology and subsequent bacteremia. Unlike our case, among the elderly and cancer patients, enterococci constitute 10-23% of bacteremic infections [6,7]. Inadequate and ineffective initial treatment has been linked to an increased mortality risk in bacteremic cholangitis - a focal point of our case, where positive outcomes were observed in our young patient with primary biliary cirrhosis.

## Case presentation

A 36-year-old male patient, who recently immigrated to the United States from Mauritania, an African country predominantly covered by desert, presented to the emergency department (ED) with a three-day history of nausea, vomiting, abdominal pain, and generalized muscle aches. The patient reported experiencing fever for the initial two days and multiple episodes of watery diarrhea, which showed improvement on the third day.

The patient's previous medical history is notable solely for an episode of jaundice five years ago. He explicitly denied experiencing any additional health concerns. Furthermore, he denied any history of drug, or alcohol use, smoking, or prior surgical procedures. Notably, his family history revealed an aunt with undiagnosed liver disease.

Physical examination findings indicated an averagely-built male patient who did not appear distressed. Jaundice was evident in the eyes and face, and pallor was observed on the tongue. The abdomen was diffusely tender without guarding, moving freely with respiration. No scars or masses were observed, and there was no organomegaly. There was no cervical or supraclavicular lymphadenopathy. Vital signs were reported as stable with blood pressure 123/84, RR 16, HR 78/min, Temp. 98.8, and EKG: 86/min, normal sinus rhythm, normal axis, and QTc of 443.

Table 1 shows the lab results at admission. CBC showed thrombocytopenia and anemia.

**Table 1:** Significant lab results: anemia WBC: White Blood Cell; RBC: Red Blood Cell; Hb: Hemoglobin; Hct: Hematocrit; MCV: Mean Corpuscular Volume; MCH: Mean Corpuscular Hemoglobin; MCHC: Mean Corpuscular Haemoglobin Concentration; RDW: Red Cell Distribution; MPV: Mean Platelet Volume

CBC	Reference Range	11/7/2023	11/8/2023	11/9/2023	11/10/2023	11/11/2023
WBC	4-5.7 10x3/µL	11	12.4	9.4	10.1	4.4
RBC	4-5.7 10x6/µL	4.2	3.5	3.8	4.8	3.7
HB	13.0-17.0 g/dl	12.8	11.8	11.8	12.1	10.1
HCT	39-53%	38.4	35.9	34.9	36.7	33.7
MCV	80-100 fL	91.4	90.6	90.1	88.8	91.1
MCH	26-33 pg	30.1	29.8	30.4	30.1	30.6
MCHC	30.5-36.0 g/dl	33	32.9	33.7	33.9	33.6
RDW	11.5-15.1%	13.5	13.3	13.3	13.9	14.6
MPV	7-11.5 fL	8.3	8.1	8.1	7.5	9.7
Platelets	130-400x3/µL	68	58	68	90	72.8

Table 2 shows the comprehensive metabolic panel (CMP) with normal electrolytes with hyperbilirubinemia, hypoalbuminemia, and acute kidney injury (AKI).

**Table 2:** Significant labs showing gradual improvement of renal functions and LFTs BUN: Blood Urea Nitrogen; CO2: Bicarbonate; ALT: Alanine Aminotransferase; AST: Aspartate Aminotransferase; Alk Phos: Alkaline Phosphatase; eGFR: Estimated Glomerular Filtration Rate

Parameters	Reference Range	11/7/2023	11/8/2023	11/09/2023	11/10/2023	11/11/2023
Glucose	70-99 mg/dl	115	113	95	111	94
BUN	7-25 mg/dl	38	37	32	16	10
Creatinine	0.7-1.3 mg/dl	2.1	2.3	1.7	1.1	1.0
Sodium	136-145 mEq/L	140	138	141	138	139
Potassium	3.5-5.1 mEq/L	3.6	2.9	3.5	3.2	3.7
Chloride	98-107 mEq/L	107	106	108	103	104
CO2	21-31 mEq/L	24	21	20	24	26
Calcium	8.6-10.3 mg/dl	7.4	7.1	7.5	7.9	7.6
Albumin	3.5-5.7 g/dl	2.8	2.3	2.4	2.7	2.5
ALT	7-52 U/L	123	26	24	29	31
AST	13-39 U/L	75	34	32	34	35
Alk Phos	34-104 U/L	119	78	76	89	112
Bilirubin	0.3-1.0 mg/dl	6.0	10.5	9.3	7.6	7.4
eGFR	>90 ml/min/173 m	33.3	41.1	36.8	52.9	89.2

Urine analysis showed proteinuria, nitrite positive, and trace leukocytes; however, urine toxicology was negative (Table 3). Moreover, the spot urine protein was 118, urine osmolality was 600, and spot urine creatine was 101.

**Table 3:** Urine analysis WBC: White Blood Cell; RBC: Red Blood Cell

Parameters	Reference Level	Results
Color	Light Yellow	Dark Orange
Urine Specific Gravity	1.005-1.030	1.025
Urine Protein	Negative mg/dl	>300
Urine Glucose	Negative mg/dl	200
Ketone	Negative mg/dl	15
Urine Bilirubin	Negative mg/dl	Large
Urine Blood	Negative mg/dl	Large
Nitrite	Negative mg/dl	Positive
Leukocytes	Negative mg/dl	Trace
WBC	0-5/HPF	Large, >20
RBC	0-4/HPF	1-4/HPF
Bacteria	None/HPF	Moderate bacteria

**Figure 1:**
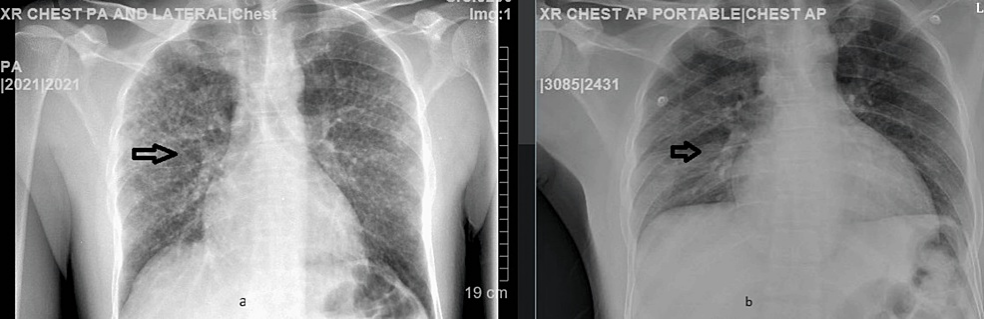
Comparison of CXR during admission and discharge a: On admission, multifocal opacities; b: On discharge, improved multifocal opacities after antimicrobial treatment CXR: Chest X-ray

Chest X-ray from admission day showed bilateral pulmonic calcifications (Figure 1) while the abdominal ultrasound (US) showed no acute pathology.

**Figure 2:**
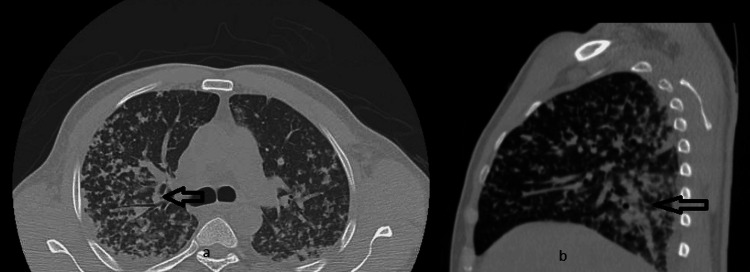
CT scan with multifocal opacities and granulomatous a: Scattered bilateral multifocal opacities in a tree-in-bud pattern; b: Represents an atypical infectious process but can also consider granulomatous changes

Computed tomography (CT) chest without contrast demonstrated scattered bilateral multifocal opacities in a tree-in-bud pattern more confluent posteriorly, along with small pleural effusions and bilateral borderline axillary adenopathy (Figure 2).

The pulmonary critical care team was consulted regarding the chest images. They recommended conducting sputum culture and gram stain, as well as tests for tuberculosis (TB) quantifiers and sputum Acid-fast bacilli. Additionally, they advised testing for urine Histoplasma antigen and immunoglobulin G (IgG) and a rheumatoid panel. A possible bronchoscopy plan was proposed if TB results were negative. Despite the comprehensive testing, all laboratory results returned negative except for the bronchoalveolar lavage (BAL), which revealed the presence of moderate Enterococcus fecalis.

**Figure 3:**
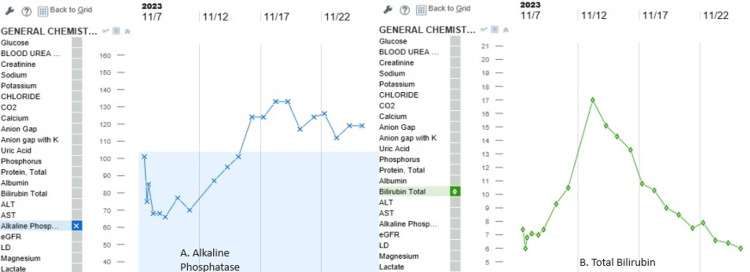
Trend of alkaline phosphatase and bilirubin a: Alkaline phosphatase stable; b: Bilirubin trending down

The anti-mitochondrial antibodies (AMA) in three samples showed increased titers. Elevated levels of alkaline phosphatase and bilirubin further supported the diagnosis of primary biliary cholangitis with pulmonary manifestations (Figure 3).

In addition, the thyroid function test, iron profile, and lipid panel were normal. Treponemal pallidum antigen (TPA) and HIV panels were also normal. Blood cultures were negative. Hb electrophoresis, glucose 6 phosphate dehydrogenase (G6PD), anti-neutrophil cytoplasmic antigen (ANCA) profile, c-ANCA (cytoplasmic), p-ANCA (perinuclear), anti-myeloperoxidase antibody (MPO) and anti-proteinase (PR3) antibodies, hepatitis panel, angiotensin-converting enzyme (ACE), carcinoembryonic antigen (CEA), and C1 esterase inhibitor were normal. Also, urine legionella, acid-fast smear, Quantiferon test, urine Histoplasma, H-pylori stool antigen, stool exam for ova, and parasites were negative. The hematology team was also consulted regarding the low platelets and anemia and they recommended CBC monitoring, peripheral blood smear for malaria, sending workup for disseminated intravascular coagulation (DIC), and gastroenterology (GI) consult. All lab results were negative and the consult team advised monitoring and medical treatment.

The patient was initially managed with hydration, empiric antibiotics, and electrolyte supplements for acute kidney injury (AKI) and gastroenteritis. Upon following up with the pulmonary critical care team, the decision was made to do a bronchoscopy for the patient. Over the next few days, chest X-ray showed partial resolution of the previously seen bilateral multifocal pneumonic infiltrates. The infectious disease team recommended continuing pneumonia antibiotic coverage with ceftriaxone and azithromycin for seven days.

## Discussion

In our case, a male patient presented with abdominal pain and PBC-related findings, including lung manifestations. In newly diagnosed cases, about half report fatigue, and one-third experience pruritus [8]. A diagnosis of PBC requires the absence of extrahepatic biliary obstruction, no liver-affecting comorbidity, and the presence of at least two of the following: alkaline phosphatase levels at least 1.5 times the upper limit of normal and antimitochondrial antibodies (AMA) at a titer of 1:40 or higher [9].

Our patient manifested symptoms of nausea, vomiting, and diffuse body aches, concomitant with evidence of direct hyperbilirubinemia. Notably, computed tomography (CT) imaging of the chest revealed bilateral multifocal opacities configured in a distinctive tree-in-bud pattern. Given the atypical nature of these presentations and the suspicion of an underlying autoimmune etiology, a detailed autoimmune workup revealed three times positive antimitochondrial antibodies with increasing titers, which confirmed the diagnosis of PBC. Several studies reveal that PBC, typically viewed as a liver-specific autoimmune disease, can also affect lung tissue. Emerging evidence suggests its potential involvement in multiple systems (liver, lung, and skin), in our case extending beyond the liver to the pulmonary system [10,11]. However, further investigations excluded sarcoidosis and silicosis, as the patient's condition was improved with antimicrobial therapy and his bronchoalveolar lavage presented with moderate enterococcus fecalis. In our case, the patient demonstrated features of sarcoidosis and lymphoid or nonspecific interstitial pneumonia or silicosis. The literature review also suggests an association exists between PBC and sarcoidosis, with some patients exhibiting features of both conditions [12,13]. In literature, this raises questions about whether they belong to a shared autoimmune spectrum or represent distinct clinical disorders with overlapping symptoms [13]. In the cohort of individuals diagnosed with PBC, it was observed that patients concurrently afflicted with interstitial lung disease (ILD) exhibited an advanced age demographic and presented with elevated levels of erythrocyte sedimentation rate (ESR) in comparison to their counterparts without ILD. This distinction reached statistical significance, as indicated by a p-value of less than 0.05 [14]. Our patient was young but had elevated ESR, which was a valuable indicator for evaluating lung function in PBC patients with spirometry. In addition, bronchoalveolar lavage (BAL) is a valuable diagnostic tool for ILD.

In our case, the bronchoalveolar lavage (BAL) fluid culture unexpectedly identified Enterococcus faecalis, initially perceived as a contaminant due to low suspicion of infection. The initial response to antibiotics raised the possibility of infection, exacerbating the underlying ILD, which improved with ceftriaxone and azithromycin. In a prospective cohort study, patients with ILD exhibited a lower prevalence of liver cirrhosis [15]. In our case, lung findings help determine less chance of liver failure and a transplant in the future as an observation from this cohort study.

Hepatic granulomas in PBC differ from those in hepatic sarcoidosis, exhibiting poor formation, non-confluence, and predominant localization within portal tracts [16]. This distinction underscores the potential utility of employing endoscopic retrograde cholangiopancreatography (ERCP) with liver biopsy as a valuable diagnostic tool for effectively differentiating between these conditions. Magnetic resonance imaging (MRI) and magnetic resonance cholangiopancreatography (MRCP) can aid in confirming clinical and laboratory findings of PBC, even in the early stages. The combined presence of parenchymal lace-like fibrosis and periportal halo sign on T2-weighted MR images demonstrated a sensitivity of 69% [17].

Our patient exhibited isolated direct hyperbilirubinemia without cirrhosis, potentially conferring a survival advantage. In primary biliary cholangitis, conjugated hyperbilirubinemia results from impaired bilirubin excretion due to obstructed bile flow, altered bile ductular integrity, or reduced bile production due to defective activity of bile efflux transporters. This raises biliary system pressure, leading to reflux of conjugated bilirubin into plasma [18,19]. Ursodeoxycholic acid is the current first-line therapy for PBC known to improve biochemical indices, delay histological progression, improve survival, and decrease the need for liver transplant. In our case, we treated the patient with antibiotics, resulting in improved liver enzymes without the necessity for additional steroids or ursodeoxycholic acid.

## Conclusions

In this case, we discussed a case presented with gastroenteritis and asymptomatic pulmonary manifestation, which was initially diagnosed as primary biliary cholangitis with interstitial lung disease. This case stands out for its unique presentation and favorable outcome. Literature suggests that PBC with pulmonary involvement poses challenges in treatment and carries increased mortality. However, our approach involved antimicrobial therapy and symptomatic management, resulting in a positive outcome. This experience provides valuable insights for future clinicians in formulating effective management plans.
